# Can Regional Eco-Efficiency Forecast the Changes in Local Public Health: Evidence Based on Statistical Learning in China

**DOI:** 10.3390/ijerph20021381

**Published:** 2023-01-12

**Authors:** Xianning Wang, Zhengang Ma, Jiusheng Chen, Jingrong Dong

**Affiliations:** 1 School of Economics and Management, Chongqing Normal University, Chongqing 401331, China; 2 Big Data Marketing Research and Applications Center, Chongqing Normal University, Chongqing 401331, China; 3 Regional Economics Applications Laboratory (REAL), University of Illinois Urbana-Champaign, Champaign, IL 61801, USA; 4 College of Life Sciences, Chongqing Normal University, Chongqing 401331, China

**Keywords:** the changes in local public health, forecasting, multiple factors, regional characteristics, statistical learning, regional eco-efficiency

## Abstract

Regional eco-efficiency affects local public health through intermediaries such as economic and environmental impacts. Considering multiple factors, the implicit and uncertain relationship with regional characteristics, and the limited data availability, this paper investigated the forecasting of changes in local public health—including the number of visits to hospitals (VTH), outpatients with emergency treatment (OWET), number of inpatients (NI), number of health examinations (NOHE), and patients discharged (PD)—using calculated regional eco-efficiency with the Least Square-Support Vector Machine-Forecasting Model and acquired empirical evidence, utilizing the province-level data in China. Results: (1) regional eco-efficiency is a good predictor in both a single and multi-factor situation; (2) the prediction accuracy for five dimensions of the changes in local public health was relatively high, and the volatility was lower and more stable throughout the whole forecasting period; and (3) regional heterogeneity, denoted by three economic and demographic factors and three medical supply and technical level factors, improved the forecasting performance. The findings are meaningful for provincial-level decision-makers in China in order for them to know the current status or trends of medical needs, optimize the allocation of medical resources in advance, and enable ample time to tackle urgent emergencies, and, finally, the findings can serve to evaluate the social effects of improving regional eco-efficiency via local enterprises or individuals and adopting sustainable development strategies.

## 1. Introduction

Economic and environmental factors affect local residents’ health, and environmental and medical decision-makers attach importance to the trends of changes in local public health (CLPH) [1]. Early prediction of the changes in local public health can provide sufficient time to balance the supply and demand of medical resources such as by making people prepared to respond to medical emergencies [2] and optimizing the resource allocation of medical materials in advance, thereby dynamically promoting a local medical service level [3]. Regional eco-efficiency can serve as a flexible indicator that integrates the relevant economic or environmental factors, those which scholars indicate can be chosen as good exogenous predictors for public health. Based on the organic performance of input and output factors [4], regional eco-efficiency (REE) has been treated as an explicitly important indicator due to it integrating both economic and environmental impacts and being closely related to healthcare sustainability [5]. Therefore, is there any empirical evidence to show REE can forecast CLPH with acceptable accuracy? 

This problem arouses many scholars’ interests in these interdisciplinary issues [6], but the literature rarely shows adequate quantitative evidence. There are three main reasons for this. First, the factors that affect residents’ health are multi-scale and multi-dimensional, including micro-level factors in individual health, macroeconomic factors, and environmental factors. Second, the description of a nonlinear relationship is uncertain, and simple linear regression cannot meet the needs of prediction. Third, the influence and heterogeneity of regional differences lead to the complexity of modeling. In the process of empirical operation, the above problems are transformed into one forecasting method with multiple factors, an implicit and uncertain relationship, regional characteristics, and limited data availability.

In the following literature review section, relevant studies have recognized and calculated the correlation between the two and the feasibility of prediction. This paper aimed to investigate this problem by building a new forecasting model based on statistical learning and obtain empirical evidence with Chinese regional data. This study can provide a new perspective and method to predict regional CLPH. In addition, in the collaborative process of promoting sustainable economic development by improving the REE, the related findings can strengthen inter- or intra-province cooperation with medical resources and improve risk management levels across different regions.

Relevant concepts about REE show that it is equipped with the capability and feasibility to forecast CLPH. As an instrument for sustainability analysis, it can reflect and judge the effectiveness of local economic activity, taking the nature of their goods or services into account [7]. Its definition, measurement, and main factors are closely associated with two kinds of forecasting variables: environmental change and economic factors. The former consists of the living conditions that directly affect the health levels of local inhabitants by impacting the air quality, water safety, solid waste disposal, and so on [8], and especially the energy efficiency and the accompanying pollutants [9]. The latter comprises the economic costs and individual financial capacities and determines the disease treatment of regional residents by impacting employment opportunities and disposable incomes. Therefore, in the economic, environmental, and medical health sciences and their intersectional fields there is inherent theoretical research or a basis to support that REE can act as one of the best leading indicators for CLPH. 

Identifying what the implicit interaction between CLPH and spatial REE is is an important prerequisite for making predictions; however, there is not a linear or convertible linear relationship because of outlier data, regional heterogeneity, the interactions of multiple influencing factors, etc. In view of the advantages of the Support Vector Machine (SVM), with its powerful identification ability within multi-dimensional complex data, it is a good choice to quickly identify the implicit relationship and accurately make predictions using the limited data sources and multiple factors.

Considering that alongside REE there are multiple factors affecting CLPH, such as an implicit and uncertain relationship, regional characteristics, and limited data availability, this paper investigated how to forecast CLPH using REE by building the Least Square-Support Vector Machine-Forecasting Model (LS-SVM-FM) and acquiring empirical evidence utilizing regional province-level data in China. Furthermore, on the bases of three forecasting error indicators, we measured the prediction accuracy for each region, such that we (1) chose VTH, OWET, NI, NOHE, and PD as proxy variables for CLPH, respectively; (2) calculated eco-efficiency with commonly required variables and collected data; (3) to reflect spatial heterogeneity, incorporated six control variables including economic and demographic factors [10] and medical supply and technical level factors; (4) compared the LS-SVM-FM without or with each control variable, respectively, and obtained the best forecasting model with a higher accuracy; (5) analyzed regional characteristics and forecasting variation in China with a comparison analysis; and (6) discussed the policy implications.

In Section 2, we conduct a literature review to have a clear understanding of existing problems for forecasting CLPH using REE. In Section 3, we present the model specification and the empirical setting of the LS-SVM-FM. Section 4 provides quantitative evidence for forecasting CLPH using REE with Chinese provincial data, and a further comparison analysis of forecasting performance is conducted. Section 5 and Section 6 show the summary and conclusion.

## 2. Literature Review

### 2.1. The Inner Relationship between CLPH and REE 

The inner relationship between CLPH and REE originates from their basic concepts. REE is a concise and simplified comprehensive index and emphasizes the monetary costs of various resources and the environmental changes required for economic development. Meanwhile, CLPH is also closely and directly associated with this kind of economic and environmental change, with regards to the requirement of maximum profits and minimum pollution. In addition, a variety of factors, such as the consumption of energy, pollutant emissions in waste gas, pollutant emissions in waste water, industrial solid wastes, the increased value of industrial development, and so on [11,12], originate from the measurement of REE and impact CLPH simultaneously. The above internal connection is an important foundation for building a predictive model, and the related literatures provide a basis for theoretical feasibility. 

(1) In the economic dimension [13,14,15,16,17], scholars have investigated how economic activities embedded in REE affect CLPH [18]. As the most basic and critical requirements, the physical health of residents necessitates food, exercise, spiritual guarantee, and so on, which are achieved under one important premise: that personal income level and economic development trends provide the fundamental roles [19]. Residents earn income through employment to purchase energy and nutrition, for continuous life and education services, etc. [20], and to afford the expenditure for necessary medical supplies, equipment, and services [21]. Macroeconomic trends serve as leading indicators for residents’ disposable incomes on the micro-level, especially for healthcare [22,23,24]. In addition, many other social or economic activities, such as city planning [25,26], immigration [27], aging [23], or housing [28], affect the changes in local public health too. In addition, REE includes the continuing impact of economic activities, and it reflects the quantitative effects from the input–output perspective. For example, producers can optimize the decision-making process of resource allocation. 

(2) In the environmental dimension [29,30], there is a variety of related literature that have probed and shown evidence that the surrounding environments incorporated into local eco-efficiency have impacted local public health. Calculations of REE already encompass environmental input–output factors, including both the BADS and GOODS [4], which have led to changes in local public health to some extent. For example, beyond a certain concentration range, the BADS, such as particulate matter (PM_2.5_) or sulfur dioxide (SO_2_), deteriorate the living environments of residents and pose a great hidden danger to public health. In particular, excessive PM_2.5_ or SO_2_ have caused many diseases of the respiratory and nervous system with both short-term and long-term damage.

Firstly, as a hot topic, public health has also been suffering from the air pollutant emissions of the manufacturing industry [31,32,33,34,35]. The highly frequent appearance of haze episodes has brought huge stress to physical and psychological health and social daily operations. Yu, Wang [36] assessed this kind of negative impact in China by using satellite observations, and Gao, Woodward [37] conducted a review of the changes in haze pollution and local public health. There are many potential risks when the concentrations are big enough. PM_10_, NO_2_, O_3_, and CO are bad for CLPH [31,38,39,40]. 

Secondly, solid or plastic waste is resistant to degradation, has low costs, and is rapidly growing, which squeezes living space and keeps deteriorating sanitary conditions [41,42,43,44] no matter what kind of waste, from economic activities or daily life. Using modified eco-efficiency indicators, Woon and Lo [45] focused on the public health and solid waste management of Hong Kong. Langdon, Chandra [46] pointed out that solid or plastic waste has led to contaminants entering the living environment. Solid or plastic waste has also caused public health to be exposed to heavy metals such as lead, mercury, cadmium, and arsenic [47,48]. Moreover, solid or plastic waste affects the growth of plants and changes in local public health [49,50], and it is not conducive to the effective prevention of toxic substances and infectious diseases and weakens the effects of health work on medical institutions and CLPH [41,51]. 

Thirdly, excessive water pollution is another important derivative that affects changes in local public health during social or economic progress [52], and, although wastewater has been purified to be utilized again [53,54], chemical compounds—toxic micropollutants—hidden in the water pollutants have gradually evolved into a huge health risk [55,56]. Saha, Rahman [57] pointed out that through ingestion or dermal contact, local residents are likely to be diseased. CLPH have continued to deteriorate and have caused various diseases due to the pesticides or toxic metals in both the irrigation and drinking water systems [58].

### 2.2. The Keys to Forecast CLPH with REE

When forecasting CLPH using REE, there are the following issues that need to be settled. (1) There are many factors that make the relationship so complex. It is necessary to adopt a new technique (SVM) to map the linear, nonlinear, or some complex implicit relationship because CLPH are influenced by economic, environmental, and individual factors, as well as others (as discussed in the last section). Simultaneously, REE with six control variables works to add to the practical interpretability. (2) There is an implicit and uncertain relationship description when using REE to forecast CLPH, especially as this paper applied five indicators as proxy variables for CLPH and six control variables. Whether they are positive or negative impacts and linear or nonlinear, this needs more quantitative evidence. (3) Different regional characteristics require a quantitative comparison of prediction performances. Considering the regional heterogeneity, it is necessary to build or estimate a model for a single region. Moreover, since there are five proxy variables for residents’ health status, there is a question worth discussing about the relatively higher prediction accuracy obtained via eco-efficiency and six other control variables. (4) There are limited sample data, and how to obtain better prediction accuracy with limited data is another question. Multiple factors and regions generally require more data to complete the fitting, obtain the optimal parameters, and further predict the data within or outside the sample on a secondary basis. It needs a strong learning ability and effective use of a small sample of information. 

## 3. Data and Methods

### 3.1. Data and Variables

**Figure 1:**
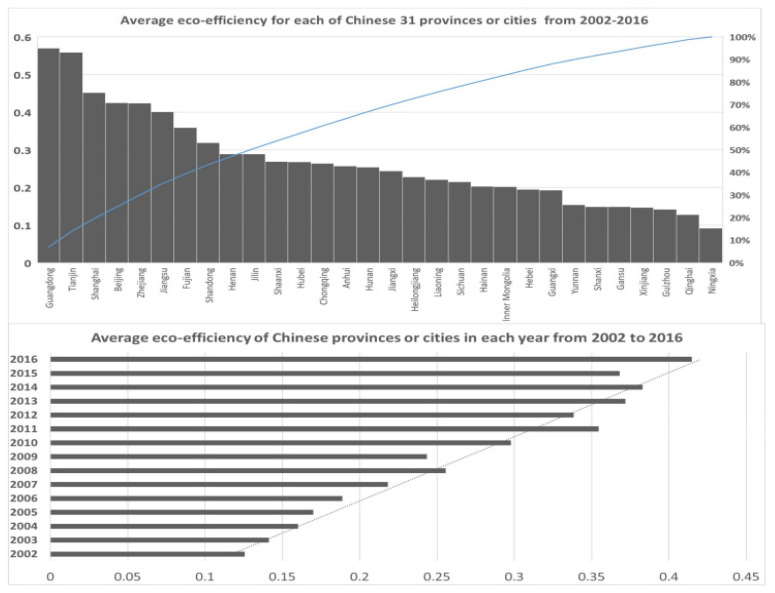
The values of REE in 30 provinces and cities of China.

Considering the data availability of and lack of data on Tibet, Hong Kong, Macao, and Taiwan, in the empirical Section 3, all provinces or cities in China were taken into account. The time period is from 2002 to 2016. This paper adopted “SBM (Slacks-Based Measure)” [59] and DEA-SOLVER Pro 5.0 [60] to measure the REEs. The descriptive statistics of the main variables to calculate the REEs are listed in Table S1 and Figure 1. The results are consistent with most other studies [61]. The eastern values of REE are higher than the western values. The value of the eco-efficiency of the whole nation is up to 0.51 in 2016.

**Figure 2:**
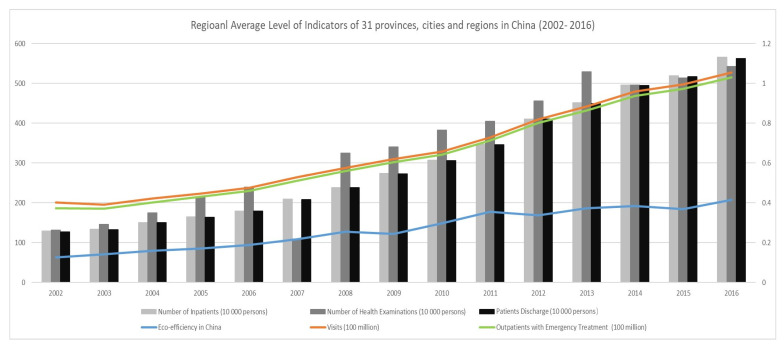
Regional average level of the used indicators in China.

The data of VTH, OWET, NI, NOHE, and PD (mainly from hospitals) are from the Chinese Medical Health Statistics Yearbooks from 2003 to 2017. The main statistics descriptions can be obtained from Figure 2. Indicators related to REE and all the control variables were mainly collected from the China Statistical Yearbooks from 2002 to 2017. All the indicators related to value were excluded because of the effect of inflation on the prices in 1998. 

In addition, due to the limitation in the same frequency processing of the data collection of the other variables in the forecasting model, the eco-efficiency calculation period is 2002–2016 [62]. First of all, it was difficult to obtain the energy-related/CO_2_-related input and output indicators (shown in Supplementary Materials: Table S1, including the main variables of the SBM to calculate the regional eco-efficiency) in some provinces and cities, such as Tibet, which limited our sample range. Secondly, there were many independent variables and dependent variables in the prediction model. This paper applied 5 indicators as proxy variables for CLPH and 6 control variables, including the development level of regional GDP, urbanization, population, and the number of local medical personnel, local licensed (assistant) doctors, and local health care institutions. In order to keep the time range of all the variables used consistent, we had to choose all data from 2002 to 2016. Although the eco-efficiency in 2017–2019 can be calculated, statistical data such as basic medical conditions (the statistical data of medical personnel, licensed doctors, or health care institutions) were scarce or had different statistical calibers, and we were limited to unifying the range of selected years. In addition, the impact of COVID-19 after 2019 can be seen as an uncertain external impact, which may need to be the focus of future research.

Figure 2 displays the regional average levels of all the used indicators in China. It can be seen that CLPH maintained a more moderate growth trend and so did the REE. However, from the theoretical explanation, they cannot be arbitrarily predicted using linear methods because there are many factors that determine the health levels of residents, for example, physical fitness, wealth, psychological factors, exercise methods, etc., which is consistent with the view in Section 2. Considering the implicit and uncertain relationship between REE and CLPH, regional characteristics, and low data availability, the following section draws on the advantages of the LS-SVM-FM in mapping and identifying the relationship (even if non-linear), which can ensure the fitting effect and prediction accuracy.

### 3.2. Method Design 

SVM performs well in building models when there are many factors or a nonlinear data pattern with small samples in many literatures, including [63,64,65,66,67]. Based on these, this paper utilized its relevant methods to ensure the fitting effect and prediction accuracy. 

(1) The implicit relationship could have a much clearer mapping in the high-dimensional hyper feature space by constructing a hyper plane and finding support vectors to represent all the information, which allowed us to predict with a small sample of data.

(2) Its diverse kernel functions (linear and nonlinear) could meet the need for complex forecasting alongside the commonly used linear models, which allowed us to predict with the complex or uncertain relationship of the forecasting model. In the high-dimensional feature space, the proposed method adopted the nonlinear kernel to map the non-linear function learned by a linear learning machine, the process of which is not limited to spatial dimensionality. 

(3) Compared with other methods, taking the “Structural Risk Minimization Principle” as the principle, SVM enabled our method to be equipped with an improved classification power [68], which allowed us to acquire a better forecasting accuracy for each region in China with a good fitting [67].

(4) Most forecasting based on SVM such as the Least Square-Support Vector Machine (LS-SVM) has already been applied to time series data, and this study extended it to regional panel data by constructing the LS-SVM-FM.

Based on the classic LS-SVM, LS-SVR, and LS-SVR-DS, this paper built the LS-SVM-FM with different regions and multiple factors.

With the dependence on the two parameters σ and  γ, the solution of the LS-SVR can be modified as the following equation:

(1) $$y\left(X;\sigma ,\gamma \right)=\overset{m}{\underset{i=1}{\sum }}\alpha_{i}\left(\sigma ,\gamma \right)K\left(x_{j},x_{i}\right)+b\left(\sigma ,\gamma \right)$$

It is better to apply the optimal method to obtain what are the true values of those main parameters by minimizing the average of squared errors. It can be displayed as

(2) $$\underset{\sigma ,\gamma }{\min}G\left(\sigma ,\gamma \right)=\frac{1}{m}\overset{m}{\underset{j=1}{\sum }}{\left[y_{j}-y\left(x_{j};\sigma ,\gamma \right)\right]}^{2}=\frac{1}{m}\overset{m}{\underset{j=1}{\sum }}{\left[y_{j}-\overset{m}{\underset{i=1}{\sum }}\alpha_{i}\left(\sigma ,\gamma \right)K\left(x_{j},x_{i}\right)-b\left(\sigma ,\gamma \right)\right]}^{2}$$

In the empirical parts, the proposed LS-SVM-FM took the CLPH as Y, whose proxy variables are, separately, the VTH, OWET, NI, NOHE, and PD. $x_{1},x_{2},x_{3}$, …, and $x_{7}$ represent variable values of REE, and all 6 of the control variables are represented as X. The next section introduces the relevant data and variables in detail. The continued LS-SVM-FM is written as the following:

(3) $$F_{p}\left(X|W\right)=Y_{p}\left(X\right)=\overset{7}{\underset{q=1}{\sum }}\overset{N}{\underset{k=1}{\sum }}{\;\alpha }_{\mathrm{pqk}}K_{p}\left(x_{q},x_{k}\right)+b_{p}$$

It rewrites as

(4) $$Y_{p}\left(X\right)=\overset{7}{\underset{q=1}{\sum }}\left[{\;\alpha }_{p1}K_{p}\left(x_{q},x_{1}\right)+{\;\alpha }_{p2}K_{p}\left(x_{q},x_{2}\right)+{\;\alpha }_{p3}K_{p}\left(x_{q},x_{3}\right)+\cdots +{\;\alpha }_{\mathrm{pN}}K_{p}\left(x_{q},x_{N}\right)\right]+b_{p}$$

p=1,…,P, where P denotes the number of regions or province or cites. Here, P is 30 and stands for the 30 provinces or cities in China. p=1,…,Q, where Q denotes the number of variables. Here, Q is 7 and stands for the 7 different variables including REE and the control variables in China. k=1,2,⋯N, where N is equal to 15, and k stands for the specific year from 2002 to 2016. There are four kinds of kernels. The Radial Basis Function (RBF) kernels $\;K\left(x,x_{k}\right)=\exp\left(-∥x-x_{k}{∥}^{2}/2\sigma^{2}\right)$ were chosen as the specific form, which has been unanimously recognized by scholars with the most frequent application, relatively [69].

### 3.3. Main Steps

Without an explicit close form on σ and  γ of G, here, we provide the following algorithm of the search procedure [69].

Step 1. Initialize a search point $B_{0}=(\sigma_{0},\gamma_{0})$ and k=1.

Step 2. Let the point, $B_{1}=(\sigma_{0}+\lambda_{\sigma },\gamma_{0}+\lambda_{\gamma })$, be alternative, in which $\lambda_{\sigma \;}$ and $\lambda_{\gamma }$ denote the random step sizes generated from the (0, 1) uniform distribution.

Step 3. Calculate $G\left(\sigma_{0},\gamma_{0}\right)$ and $G\left(\sigma_{0}+\lambda_{\sigma },\gamma_{0}+\lambda_{\gamma }\right)$ simultaneously by applying (2).

Step 4. Replace $\sigma_{0}$ with $\sigma_{0}+\lambda_{\sigma }$ and $\gamma_{0}$ with $\gamma_{0}+\lambda_{\gamma }$, if $G\left(\sigma_{0}+\lambda_{\sigma },\gamma_{0}+\lambda_{\gamma }\right)\leq G\left(\sigma_{0},\gamma_{0}\right)$. Otherwise, $\sigma_{0}=\sigma_{0}$ and $\gamma_{0}=\gamma_{0}$.

Step 5. When $G\left(\sigma_{0},\gamma_{0}\right)\leq \varepsilon$ or  k≥N, the iteration can stop. Otherwise, set k≥k+1 and return to Step 2. The iteration can stop either when the forecasting accuracy can be achieved or the computation is finished within an exogenously prespecified iteration number  N. When the algorithm stops, it finds the ‘optimal’ pair of $\left(\sigma_{0},\gamma_{0}\right)$ for the LS-SVM-FM, which minimizes the training error.

The main procedures are described as follows: (1) We applied each of the 30 province-level datasets in China to the LS-SVM-FM and performed in-sample learning and fitting to determine the parameter value and out-of-sample prediction and comparison to determine the prediction accuracy; (2) mean percentage error (MPE) and mean square or standard deviation of prediction error (MSE or SDE) were chosen to judge the prediction accuracy; (3) the VTH, OWET, NI, NOHE, and PD for CLPH were respectively taken as Y; (4) REE and the control variables in China were adopted as X, and the LS-SVM-FM without or with each of the 6 control variables were compared, respectively, and we obtained the best forecasting model with a lower prediction error; and (5) the forecasting accuracy with the single factor and multiple factors in China was drawn from the comparison analysis [69].

(1) $\mathrm{MPE}=\frac{\sum_{t=1}^{T}\frac{y_{t}-\hat{y_{t}}}{y_{t}}}{T}$.
(2) $\mathrm{MSE}=\frac{\sum_{t=1}^{T}{\left(y_{t}-\hat{y_{t}}\right)}^{2}}{T}$; $\mathrm{SDE}=\sqrt{\frac{\sum_{t=1}^{T}{\left(y_{t}-\hat{y_{t}}\right)}^{2}}{T}}$.

$y_{T}$ and $y_{t}$ denote the known sample values of the year t. $\hat{y_{T}}$ and $\hat{y_{t}}$ denote the predicted sample values of the last year and the year of t using the LS-SVM-FM. t=1,2,⋯,T.

## 4. Results

### 4.1. Forecasting Accuracy with the Single Factor and Multiple Factors

Chinese data were applied to the LS-SVM-FM to obtain empirical evidence. To understand the prediction better and keep a reasonable explanation, each of the five proxy indicators of CLPH for the thirty different provinces and cities in China were forecasted, and we compared the prediction errors from the following three aspects: (I) utilizing the single-factor-REE to forecast the CLPH in China, naming the model LS-SVM-FM (1); (II) adopting the multi-factors-REE and three more economic and demographic factors, naming the model LS-SVM-FM (2); and (III) based on the LS-SVM-FM (2), incorporating three more medical supply and technical level factors, naming the model LS-SVM-FM (3).

The three models obviously showed whether the forecasting accuracy changed significantly when applied to more control variables and which model gained the lowest prediction errors of each selected region in China. Table 1, Table 2, Table 3, Table 4, Table 5 and Table 6 display detailed corresponding information about the forecasting performances of LS-SVM-FM (1), LS-SVM-FM (2), and LS-SVM-FM (3).

When only using the REE to forecast the CLPH in China, performance comparisons within Table 1 and Table 2 provide more information. Firstly, overall, REE can better predict the health status of regional residents of the five proxy variables in China. The MPE values all fall within the acceptable interval, and in particular, the average MPE values of the CLPH are 1.295%, 1.235%, 2.961%, 16.028%, and 2.985%, although the NOHE is bigger than 10%, and 12 of the 30 regions show a bigger than 10% prediction error. As with the literature mentioned above, REE owned the impacts from both economic and environmental aspects at the same time, and it is crucial and well-behaved for describing the health conditions of residents. Change in eco-efficiency affect living conditions and thus the changes in local public health. Therefore, it can be regarded as a good predictor, helping decision-makers to quantify future changes in residents’ health in advance and, finally, adjust various medical supplies and technical preparations.

Secondly, the volatility of the forecasting error appears quite differently in each province or city, as represented by the bigger values of MSE and SDE for NI, NOHE, and PD compared with the other indicators. Their bigger MSE values are in part due to a smaller statistical unit, but SDE is much more convincing, with values of 128.99, 70.52, and 33.00. Another possibility is whether the model ignores important explanatory variables or other observed factors because local public health can actually be impacted by a number of factors no matter if on the individual or environmental level, as analyzed in the previous literature review.

Thirdly, regardless of the vertical comparison of a resident’s health status or the horizontal comparison of different indicators, significant differences in forecasting accuracy between provinces and cities also exist, or the influence of regional heterogeneity on prediction accuracy is very obvious. Regional decision-makers should notice the phenomenon. The specific situations of different provinces or cities are important clues for analyzing the above differences of LS-SVM-FM (1).

By adding the relevant control variables to consider the multiple factors of the LS-SVM-FM at two times, it helps to understand the aspects confirmed in the above analysis. Table 3 and Table 4 shows the forecasting performance of LS-SVM-FM (2), with REE and GDP per capita, urbanization level, population density; Table 5 and Table 6 shows the forecasting performance of LS-SVM-FM (3), with medical supply and technical level factors, based on the former one. As presented in Table 3, Table 4, Table 5 and Table 6, other information on economic population and supplementary information on medical supply and technical level helps to better understand the potential relationship between CLPH and REE in China when constructing an empirical forecasting model.

Firstly, incorporating the six control variables enables the model to obtain better results. LS-SVM-FM (3) and LS-SVM-FM (2) show better or higher forecasting accuracies than LS-SVM-FM (1). LS-SVM-FM (3) is the best one based on all the values of MPE, MSE, and SDE for CLPH in most provinces or cities in China. For example, the minimum averages of MPE are 0.06%, 0.05% 0.12%, 13.72%, and 0.08% and as are the values of MSE and SDE. This can be attributed to the control variables to provide better information for machine learning methods in order to identify more realistic mapping relationships in high-dimensional spaces.

Secondly, the main findings in Table 3, Table 4, Table 5 and Table 6 present similar results as those in Table 1 and Table 2. The other reason for the overall continuously improved prediction effect is that the radial basis kernel function better describes the above relationship, and it can take into account the linear and nonlinear relationships between multiple explanatory variables to the greatest possible extent. The 7 explanatory variables (eco-efficiency and 6 control variables) and the one-to-one regression for the 30 selected regions of China established high requirements for the sample size. Because there are only 15 years of data, the traditional panel model fitting and prediction effects were limited. However, the method proposed in this paper only needs to find the support vectors due to the advantage of the conversion of the high-dimensional space, but with extra data or information still needed. At the same time, the powerful calculation and learning capabilities make up for the limited data.

Thirdly, the NOHE of CLPH owns the bigger prediction errors for MPE, MSE, and DSE in the three models than the other four. Some points can explain some of the reasons. For example, the raw data of health examinations fluctuated greatly in 2007, especially in the Shaanxi Province. In addition, as well as the factors already considered, the health examinations may be related to the medical insurance system in China and medical process of medical and health institutions, and further research is required.

### 4.2. Forecasting Variation with the Single Factor and Multiple Factors

The previous section gave specific prediction accuracies and a corresponding direct analysis. However, when actually predicting the CLPH, in addition to the annual forecast performance and change, scholars also arouse attention to how the changes in forecast errors shift across the time dimension, including changes in averages (average degree) and changes in variance (variation degree), that is to say, how the MPE, MSE, and SDE change according to time. At the province level, it was shown that the values of the averages and standard deviations of MPE, MSE, and SDE for each of the five proxy variables of the changes in local public health levels in China. Furthermore, it can be learned that the concentration trend and degree of dispersion of the forecast error change, based on which the reliability and robustness of the models can be analyzed.

As is shown in Table 5 and Table 6, for the forecasting variation of the LS-SVM-FM in China, LS-SVM-FM (3) outperformed LS-SVM-FM (1) and LS-SVM-FM (2) with the greatest number of average degrees and variation degrees for all five variables of the CLPH. The minimum value of each line is marked with bold font, which represents the overall variation in the forecast error at the province or city level. The prediction accuracies of some provinces or cities are very high, and for some others are very small, but the overall prediction accuracy is acceptable for LS-SVM-FM (1), LS-SVM-FM (2), and LS-SVM-FM (3). Taking LS-SVM-FM (3) as an example, the lowest value of average degree is about 0.00060 (change in MPE) for VTH of CLPH, 0.00012 (change in MSE) for OWET, and 0.007099 (change in SDE) for OWET, and the lowest values of variation degree are about 0.00061 (change in MPE) for OWET, 0.00032 (change in MSE) for OWET, and 0.00808 (change in SDE) for OWET.

By comparing the average degrees and variation degrees of prediction errors such as MPE, MSE, and SDE, from the global perspective, LS-SVM-FM (3), taking into account all six control variables, was more reliable and has a higher relative robustness than LS-SVM-FM (1) and LS-SVM-FM (2), although the other two models are also acceptable within a certain range of prediction accuracy. Meanwhile, the forecast volatility of NI, NOHE, and PD significantly expanded, so it is the best choice to make short-term or spot predictions on the above three dimensions of the CLPH.

## 5. Discussion

### 5.1. Main Revelation

REE affects the CLPH through intermediaries that are integrated and represented by the economic and environmental impacts from the inputs or outputs of computing the REE at the provincial level in China. Specifically speaking, green sustainable development is to improve eco-efficiency and encourage consumers, producers, and managers to avoid excessive pollution [12]. The continuous increase in green behaviors in work and life has improved the living environment on which residents depend. Under the premise of ensuring environmental protection, economic achievements improve the disposable income for living standards and medical conditions and reduce pollution in living environments and guarantee a reduction in disease.

The calculated prediction results can be used as the basis for evaluating the specific social effects of adopting sustainable development strategies by local enterprises or individuals. Making residents’ living or health conditions better is one of the most fundamental pursuits of a higher REE in each province or city. Local inhabitants are the ultimate maintainers and beneficiaries. Therefore, empirical findings by forecasting CLPH via REE could serve as a tool to evaluate the performance of REE-related policy formulation and activity implementation and find out the actual effects and deficiencies that need to be addressed to guide sustainable development practices.

The innate differences and respective characteristics between provinces in China are an important material for explaining the imbalance of spatial medical demand and supply distribution. For example, there are three economic and demographic factors and three medical supply and technical level factors to reflect regional heterogeneity. These factors show why the real situations of CLPH and the magnitudes of change are different across different provinces, and the above six factors explain the different effects of increasing REE promotion on local CLPH, although there are other individual, behavioral, climatic, psychological, and even political factors for CLPH such as nutrition, climate change, noise, institutional determinants, medical insurance, and so on. However, considering the quite limited data availability from micro-individual statistics, it is necessary to investigate what the interactions are between the economy, environment, and local public health at the overall macro- and meso-levels.

### 5.2. Policy Implications

To be more specific, this study is very helpful for decision-makers in each province of China to understand and optimize the allocation of medical resources. With the help of the early information on CLPH obtained with the right proposed model, which can forecast VTH, OWET, NI, NOHE, and PD with a high prediction accuracy in 30 provinces or cities of China, decision-makers can take this as the quantization basis to confront some urgent emergencies via a continuous supply of medical supplies. It is an important guarantee for changes in local public health.

**Figure 3:**
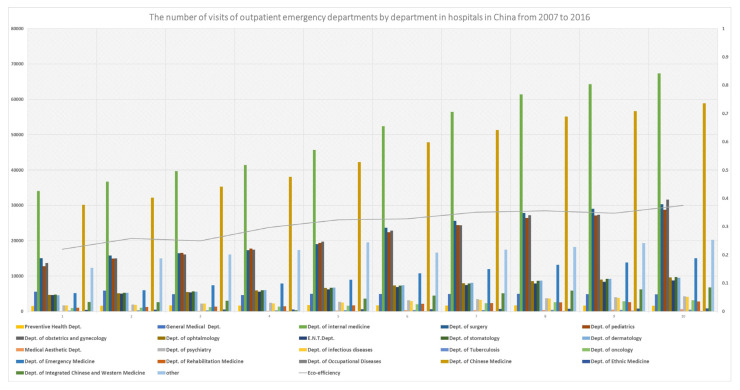
Comparison by department of visits to outpatient emergency departments in China.

Taking VTH as an example, in addition to the general medical supplies, different departments of VTH require independent professionals and medical resources, and more advanced forecasts provide time and a quantitative basis for the production, purchase, and storage of various medicines, disinfectants, or medical tools, from a general point of view. Combining the whole forecast for VTH with the ratios of all the sub-departments on average, there will be more evidence to distinguish the most important demand or emergency, and the expensive medical supplies to be purchased from others. As it is shown in Figure 3, and Figures S1 and S2, the Departments of Internal Medicine, Chinese Medicine, Surgery, Obstetrics and Gynecology, and Pediatrics ranked in the top five, which reflects the differentiated needs and the five most common problems in residents’ health. It seems obvious that the medical resources required by the five departments vary greatly. The treatment methods of the Department of Chinese Medicine have more Chinese characteristics. The medicines are concentrated in Chinese herbal medicines. The production and use of medicines and rehabilitation training require special medical equipment (acupuncture equipment, medical Tuina, etc.). In addition, the requirements for medical equipment for testing, diagnosis, or even treatment between Departments of Internal Medicine and Surgery vary widely with a higher accuracy. The Department of Pediatrics have higher requirements for the various ingredients of drugs, which are different from those for adults, as are the mentioned medical staff and job requirements in different professional directions. Therefore, these top five need to be given enough attention according to the real conditions of the 30 selected regions in China, and plans should be made about the following aspects, including enough medical workers, prepared medical resources, and earlier cooperation with upstream and downstream enterprises.

From the perspective of risk and early warning, related forecasting results can be used as an important reference for early warning and risk identification in public health. Taking VTH as an example, more detailed comparisons of different sub-departments between regions are attached in the Supplementary Material. Obtaining the regional heterogeneity of CLPH in the figures through our predictions, based on the perspectives of local residents’ eating habits, disposable incomes, and population density, decision-makers can formulate local medical material reserve methods and emergency medical incident response plans. As the figures show, for the top five sub-departments with high numbers of visits that are urgently needed in various regions, policy or tax support can be provided to promote the healthy development of the related industries in the long run. For short-term fluctuations in individual provinces or cities, certain consultation or coordination mechanisms can be adopted between other regions to deploy medical personnel and materials to increase efficiency and reduce the waste of resources, just as that in Guangdong, Shandong, and Shanxi. As a whole, forecasting results from the other four indicators—OWET, NI, NOHE, and PD—can also be utilized as with the above analysis with some specific auxiliary information. Accurate predictions from the above five dimensions can help to detect residents’ medical conditions. The primary advantage of the above results is that they can optimize medical supplies and personnel in various regions in time and grasp the overall situation of different types of medical needs. For example, according to the need changes in OWET, inpatients, health examinations, and patients discharged, the medical industry can dynamically adjust the supply and reserves of materials, reduce inventory, and minimize waste and excessive use of medical resources, and especially important medicines and instruments that are in short supply and have a long production cycle.

From the perspective of decision optimization, through findings on the control variables and how to calculate the required indicators of regional eco-efficiency, we can learn a differentiated path to improve public health in different regions. When adopting economic policies and measures for local sustainable development for improving the REE, they should consider regional differences and be possible to adjust to the most urgent and corresponding factors that affect CLPH in real-time, according to their own economic development level. Furthermore, it is helpful to strengthen regional cooperation to optimize the allocation of medical resources. These main findings can guide the industry or the government to strengthen the close cooperation between upstream and downstream enterprises in the medical industry. Accurate forecasting guarantees that there is enough time to carry out the following work: technical cooperation that breaks through key technical bottlenecks, resource coordination that reduces overall risks, and personnel exchanges that share prevention experience.

## 6. Conclusions

### 6.1. Main Findings

REE is a highly synthetic indicator with integrated economic and environmental impacts that is associated with local CLPH. Considering that there are multiple factors affecting CLPH in addition to REE, such as the implicit and uncertain relationship between the two, regional characteristics, and low data availability, this paper investigated how to forecast CLPH using REE by utilizing the LR-SVM-FM and acquire empirical evidence utilizing the regional province-level data in China.

Taking REE as the main predictor and province-level data in China, this paper investigated how five proxy variables of CLPH were predicted, with different control variables including more economic and demographic factors and three more medical supply and technical level factors. Some interesting empirical findings were that (1) REE is a good predictor for predicting residents’ health, whether in a single-factor situation or a multi-factor situation. (2) The proxy indicators that measure the health status of residents have different prediction effects. The prediction accuracy of VTH, OWET, and NI is relatively high and the volatility is lower and more stable throughout the whole forecasting period. (3) Utilizing three economic and demographic factors and three medical supply and technical level factors can improve forecasting performance. (4) The LR-SVM-FM based on machine learning meets the forecasting needs: regional heterogeneity of provinces and cities in China, limited samples, uncertain functional relationships, etc.

As explained and proposed earlier, the results show that (1) REE is a comprehensive indicator that combines the dual impacts of the economy and the environment, which are also important factors that affect residents’ health conditions. (2) The proposed prediction model relying on the machine learning method can better characterize the uncertain and complex relationship between different regions and multiple influencing factors with limited samples. (3) Six control variables from economic factors, technical factors, and demographic factors improve the model with a higher degree of explanation, which is more in line with the real phenomenon.

### 6.2. Future Research

This article tried to conduct interdisciplinary research by forecasting CLPH using regional eco-efficiency with integrated economic and environmental impacts. Future research based on this could include searching for more micro-individuals and psychological indicators, or happiness indexes, to improve prediction models; quantifying the impacts of regional interactions on the prediction effect; and understanding how medical emergencies and responses to them can be influenced by the prediction performance.

## Figures and Tables

**Table 1:** The First Part of Forecasting performance of LS-SVM-FM (1).

	Visits (100 Million)			Outpatients with Emergency Treatment (100 Million)			Number of Inpatients (10,000 Persons)		
	MPE	MSE	SDE	MPE	MSE	SDE	MPE	MSE	SDE
China	0.00192	0.91664	0.95741	0.00203	0.90207	0.94977	0.00377	405,619.57361	2466.63609
Beijing	0.00593	0.00166	0.04068	0.00413	0.00138	0.03716	0.00641	55.98069	28.97776
Tianjin	0.00347	0.00059	0.02431	0.00370	0.00060	0.02449	0.00429	23.26127	18.67937
Hebei	0.00621	0.00550	0.07414	0.00684	0.00532	0.07292	0.00596	1922.83207	169.83074
Shanxi	0.03625	0.00497	0.07052	0.03664	0.00439	0.06627	0.08777	4054.84798	246.62263
Inner Mongolia	0.01775	0.00161	0.04012	0.01747	0.00149	0.03857	0.01914	540.89685	90.07471
Liaoning	0.01685	0.00877	0.09366	0.01881	0.00904	0.09510	0.04576	7055.55512	325.32034
Jilin	0.00218	0.00019	0.01373	0.00178	0.00016	0.01264	0.00206	76.35504	33.84266
Heilongjiang	0.05188	0.01065	0.10321	0.05657	0.01089	0.10435	0.14120	10,230.06615	391.72821
Shanghai	0.00971	0.00575	0.07583	0.01049	0.00576	0.07593	0.00921	310.72021	68.27007
Jiangsu	0.00149	0.00193	0.04392	0.00248	0.00196	0.04428	0.00294	265.18282	63.06935
Zhejiang	0.00311	0.00343	0.05856	0.00330	0.00342	0.05845	0.00457	409.03585	78.32967
Anhui	0.00299	0.00124	0.03519	0.00207	0.00112	0.03340	0.00349	662.15291	99.66089
Fujian	0.00818	0.00237	0.04869	0.01005	0.00244	0.04940	0.01062	710.07202	103.20407
Jiangxi	0.00194	0.00036	0.01898	0.00191	0.00034	0.01834	0.00432	327.05850	70.04197
Shandong	0.00302	0.00569	0.07545	0.00382	0.00554	0.07442	0.00569	3580.87560	231.76094
Henan	0.00197	0.00172	0.04151	0.00126	0.00154	0.03920	0.00328	985.31963	121.57218
Hubei	0.00218	0.00055	0.02341	0.00158	0.00040	0.02000	0.00338	233.81192	59.22144
Hunan	0.00112	0.00053	0.02300	0.00111	0.00048	0.02195	0.00459	492.60320	85.95957
Guangdong	0.00243	0.01016	0.10081	0.00249	0.00987	0.09933	0.00309	1056.46133	125.88455
Guangxi	0.00156	0.00039	0.01964	0.00093	0.00031	0.01770	0.00610	299.17792	66.99006
Hainan	0.05362	0.00068	0.02611	0.05283	0.00066	0.02564	0.11745	292.27729	66.21298
Chongqing	0.00259	0.00033	0.01826	0.00177	0.00026	0.01622	0.00927	272.60830	63.94626
Sichuan	0.00290	0.00081	0.02839	0.00303	0.00076	0.02759	0.00086	520.90749	88.39464
Guizhou	0.00306	0.00011	0.01027	0.00587	0.00030	0.01744	0.01692	471.29101	84.07952
Yunnan	0.01100	0.00542	0.07364	0.01093	0.00509	0.07136	0.01956	3485.27203	228.64619
Shaanxi	0.01456	0.00387	0.06220	0.01480	0.00382	0.06181	0.03167	3495.36887	228.97715
Gansu	0.02359	0.00188	0.04336	0.02318	0.00164	0.04048	0.10303	3128.21615	216.61773
Qinghai	0.02693	0.00019	0.01381	0.03077	0.00017	0.01296	0.06899	148.65021	47.22026
Ningxia	0.00420	0.00006	0.00765	0.00435	0.00005	0.00731	0.00751	20.19197	17.40344
Xinjiang	0.04769	0.00653	0.08080	0.05348	0.00660	0.08124	0.13928	8130.00502	349.21351
**Average**	**0.01235**	**0.00293**	**0.04633**	**0.01295**	**0.00286**	**0.04553**	**0.02961**	**1775.23518**	**128.99176**

**Table 2:** The Second Part of Forecasting performance of LS-SVM-FM (1).

	Number of Health Examinations (10,000 Persons)			Patients Discharged (10,000 Persons)		
	MPE	MSE	SDE	MPE	MSE	SDE
China	0.05315	1,738,096.51187	1318.36888	0.00379	468,007.97061	684.11108
Beijing	0.05275	2321.08266	48.17762	0.00584	56.57502	7.52164
Tianjin	0.00583	54.18368	7.36096	0.00440	23.06877	4.80300
Hebei	0.10931	4276.50913	65.39502	0.00584	1830.36096	42.78272
Shanxi	1.16767	277,966.67011	527.22545	0.08932	3988.56250	63.15507
Inner Mongolia	0.09933	298.63334	17.28101	0.01811	510.15312	22.58657
Liaoning	0.03394	2655.96989	51.53610	0.04458	6920.06978	83.18696
Jilin	0.00325	21.97031	4.68725	0.00128	69.38246	8.32961
Heilongjiang	0.34022	6837.08484	82.68667	0.14290	10,187.72469	100.93426
Shanghai	0.35247	5197.56606	72.09415	0.00811	301.01225	17.34970
Jiangsu	0.54699	17,129.53347	130.87984	0.00296	281.25033	16.77052
Zhejiang	0.58323	20,354.73403	142.67002	0.00292	311.05583	17.63677
Anhui	0.09713	2662.96101	51.60389	0.00470	682.88916	26.13215
Fujian	0.11796	2643.99917	51.41983	0.01340	669.29284	25.87069
Jiangxi	0.00329	83.20221	9.12152	0.00469	329.00479	18.13849
Shandong	0.05606	10,654.74975	103.22185	0.00566	3597.07065	59.97558
Henan	0.08149	5035.28206	70.95972	0.00371	1019.82336	31.93467
Hubei	0.02445	1362.81613	36.91634	0.00401	247.69873	15.73845
Hunan	0.00602	433.10634	20.81121	0.00443	477.56503	21.85326
Guangdong	0.17755	75,738.38389	275.20608	0.00369	1185.90951	34.43704
Guangxi	0.02221	959.26509	30.97200	0.00543	292.49687	17.10254
Hainan	0.23382	79.71844	8.92852	0.11625	291.26718	17.06655
Chongqing	0.01628	300.69067	17.34043	0.00860	258.00590	16.06256
Sichuan	0.00255	198.86296	14.10188	0.00544	378.55165	19.45640
Guizhou	0.03541	727.41516	26.97064	0.01709	455.62373	21.34534
Yunnan	0.01910	1054.97890	32.48044	0.01991	3483.63044	59.02229
Shaanxi	0.15467	3689.90557	60.74459	0.03075	3476.96806	58.96582
Gansu	0.03230	649.66192	25.48847	0.10175	3061.53453	55.33114
Qinghai	0.10173	63.68169	7.98008	0.07157	152.00172	12.32890
Ningxia	0.02544	48.12243	6.93703	0.00763	19.27320	4.39013
Xinjiang	0.30604	13,059.15345	114.27665	0.14053	8078.00936	89.87775
**Average**	**0.16028**	**15,218.66314**	**70.51584**	**0.02985**	**1754.52775**	**33.00289**

**Table 3:** The First Part of Forecasting performance of LS-SVM-FM (2).

	Visits (100 Million)			Outpatients with Emergency Treatment (100 Million)			Number of Inpatients (10,000 Persons)		
	MPE	MSE	SDE	MPE	MSE	SDE	MPE	MSE	SDE
China	0.00035	0.03927	0.19816	0.00034	0.03387	0.18404	0.00048	18,889.43367	137.43884
Beijing	0.00338	0.00092	0.11736	0.00212	0.00058	0.02415	0.00240	32.24121	5.67813
Tianjin	0.00039	0.00002	0.01598	0.00038	0.00002	0.00399	0.00000	0.00000	0.00003
Hebei	0.00003	0.00000	0.00344	0.00000	0.00000	0.00017	0.00072	91.60878	9.57125
Shanxi	0.00148	0.00010	0.03899	0.00133	0.00009	0.00935	0.00013	1.21191	1.10087
Inner Mongolia	0.00114	0.00003	0.02198	0.00090	0.00002	0.00470	0.00079	13.90730	3.72925
Liaoning	0.00001	0.00000	0.00209	0.00000	0.00000	0.00002	0.00000	0.00000	0.00020
Jilin	0.00006	0.00000	0.00601	0.00068	0.00004	0.00641	0.00059	11.04432	3.32330
Heilongjiang	0.00000	0.00000	0.00000	0.00017	0.00002	0.00421	0.00039	21.98675	4.68900
Shanghai	0.00020	0.00005	0.02828	0.00023	0.00005	0.00740	0.00022	3.04555	1.74515
Jiangsu	0.00043	0.00057	0.09235	0.00072	0.00071	0.02667	0.00006	15.07263	3.88235
Zhejiang	0.00188	0.00136	0.14269	0.00218	0.00143	0.03786	0.00161	158.65937	12.59601
Anhui	0.00093	0.00009	0.03632	0.00063	0.00005	0.00690	0.00011	15.50991	3.93826
Fujian	0.00024	0.00004	0.02592	0.00022	0.00002	0.00499	0.00222	42.85782	6.54659
Jiangxi	0.00095	0.00007	0.03204	0.00085	0.00006	0.00758	0.00000	0.00000	0.00010
Shandong	0.00106	0.00086	0.11340	0.00126	0.00074	0.02718	0.00110	409.18927	20.22843
Henan	0.00031	0.00016	0.04969	0.00000	0.00000	0.00063	0.00105	189.65446	13.77151
Hubei	0.00114	0.00027	0.06360	0.00114	0.00026	0.01603	0.00293	196.14484	14.00517
Hunan	0.00004	0.00000	0.00752	0.00000	0.00000	0.00000	0.00022	23.05697	4.80177
Guangdong	0.00064	0.00265	0.19935	0.00061	0.00258	0.05083	0.00029	506.50071	22.50557
Guangxi	0.00000	0.00000	0.00000	0.00085	0.00013	0.01125	0.00042	45.89589	6.77465
Hainan	0.00010	0.00000	0.00300	0.00008	0.00000	0.00087	0.00081	0.72074	0.84896
Chongqing	0.00032	0.00001	0.01292	0.00102	0.00003	0.00591	0.00124	8.16018	2.85660
Sichuan	0.00076	0.00034	0.07123	0.00073	0.00030	0.01718	0.00094	240.77377	15.51689
Guizhou	0.00213	0.00009	0.03756	0.00124	0.00005	0.00681	0.00862	178.35468	13.35495
Yunnan	0.00015	0.00003	0.01959	0.00020	0.00003	0.00566	0.00065	46.15658	6.79386
Shaanxi	0.00026	0.00003	0.02008	0.00020	0.00002	0.00396	0.00013	1.56745	1.25198
Gansu	0.00025	0.00001	0.01051	0.00023	0.00001	0.00258	0.00286	19.70234	4.43873
Qinghai	0.00217	0.00001	0.00961	0.00340	0.00001	0.00295	0.00988	6.72635	2.59352
Ningxia	0.00064	0.00000	0.00833	0.00042	0.00000	0.00154	0.00027	0.18873	0.43443
Xinjiang	0.00016	0.00000	0.00800	0.00026	0.00002	0.00428	0.00006	0.16210	0.40262
**Average**	**0.00071**	**0.00026**	**0.03993**	**0.00074**	**0.00024**	**0.01007**	**0.00136**	**76.00335**	**6.24600**

**Table 4:** The Second Part of Forecasting performance of LS-SVM-FM (2).

	Number of Health Examinations (10,000 Persons)			Patients Discharged (10,000 Persons)		
	MPE	MSE	SDE	MPE	MSE	SDE
China	0.05708	1,675,850.37137	1294.54640	0.00054	21,295.74204	145.93061
Beijing	0.05268	2135.02766	46.20636	0.00242	24.89391	4.98938
Tianjin	0.00233	9.22267	3.03688	0.00000	0.00000	0.00001
Hebei	0.10389	3419.73632	58.47851	0.00096	99.69114	9.98455
Shanxi	0.98408	262,613.20253	512.45800	0.00009	0.65840	0.81142
Inner Mongolia	0.22479	701.77480	26.49103	0.00062	9.30115	3.04978
Liaoning	0.01316	1020.55332	31.94610	0.00000	0.00000	0.00003
Jilin	0.05828	302.46745	17.39159	0.00039	9.07593	3.01263
Heilongjiang	0.15970	1494.46364	38.65829	0.00028	18.70821	4.32530
Shanghai	0.33256	4048.37308	63.62683	0.00028	3.82234	1.95508
Jiangsu	0.57970	18,005.27888	134.18375	0.00010	21.60201	4.64780
Zhejiang	0.58310	18,751.62824	136.93658	0.00155	98.13550	9.90634
Anhui	0.10308	2622.21053	51.20752	0.00015	23.27889	4.82482
Fujian	0.09563	1881.63866	43.37786	0.00155	39.77409	6.30667
Jiangxi	0.02288	406.57600	20.16373	0.00094	18.46146	4.29668
Shandong	0.05166	8354.16534	91.40112	0.00063	407.54852	20.18783
Henan	0.01490	627.91876	25.05831	0.00107	183.87315	13.55998
Hubei	0.02629	1359.24663	36.86796	0.00332	218.08177	14.76759
Hunan	0.00733	371.56303	19.27597	0.00021	22.10366	4.70145
Guangdong	0.19417	83,228.00416	288.49264	0.00112	348.43544	18.66643
Guangxi	0.01360	526.90583	22.95443	0.00000	0.00000	0.00009
Hainan	0.22777	46.11755	6.79099	0.00078	0.74638	0.86393
Chongqing	0.02990	475.66845	21.80982	0.00103	7.68410	2.77202
Sichuan	0.02201	1746.69817	41.79352	0.00093	226.88005	15.06254
Guizhou	0.02494	450.87739	21.23387	0.00813	168.95577	12.99830
Yunnan	0.00770	345.33049	18.58307	0.00084	55.21420	7.43063
Shaanxi	0.00000	0.00000	0.00057	0.00013	1.65379	1.28600
Gansu	0.01446	173.65754	13.17792	0.00294	22.16623	4.70810
Qinghai	0.07494	26.13551	5.11229	0.00587	2.30415	1.51794
Ningxia	0.00038	0.26050	0.51040	0.00028	0.21509	0.46378
Xinjiang	0.05055	1352.35582	36.77439	0.00000	0.00000	0.00014
**Average**	**0.13588**	**13,883.23530**	**61.13334**	**0.00122**	**67.77551**	**5.90324**

**Table 5:** The First Part of Forecasting performance of LS-SVM-FM (3).

	Visits (100 Million)			Outpatients with Emergency Treatment (100 Million)			Number of Inpatients (10,000 Persons)		
	MPE	MSE	SDE	MPE	MSE	SDE	MPE	MSE	SDE
China	0.00008	0.01127	0.10614	0.00005	0.00699	0.08359	0.00027	28,446.81852	168.66185
Beijing	0.00338	0.00086	0.02939	0.00256	0.00056	0.02358	0.00208	26.96360	5.19265
Tianjin	0.00055	0.00004	0.00655	0.00047	0.00003	0.00576	0.00025	1.61696	1.27160
Hebei	0.00080	0.00017	0.01313	0.00024	0.00005	0.00674	0.00119	106.43587	10.31678
Shanxi	0.00003	0.00000	0.00023	0.00012	0.00000	0.00072	0.00012	28.64374	5.35198
Inner Mongolia	0.00148	0.00004	0.00592	0.00129	0.00003	0.00512	0.00059	11.76455	3.42995
Liaoning	0.00036	0.00009	0.00928	0.00030	0.00010	0.01004	0.00030	40.62011	6.37339
Jilin	0.00061	0.00003	0.00555	0.00136	0.00006	0.00795	0.00113	14.20003	3.76829
Heilongjiang	0.00000	0.00000	0.00000	0.00000	0.00000	0.00002	0.00226	86.19048	9.28388
Shanghai	0.00006	0.00003	0.00564	0.00005	0.00003	0.00535	0.00006	2.09720	1.44817
Jiangsu	0.00000	0.00000	0.00000	0.00000	0.00000	0.00021	0.00010	2.58493	1.60777
Zhejiang	0.00009	0.00008	0.00867	0.00011	0.00009	0.00944	0.00019	13.05925	3.61376
Anhui	0.00016	0.00002	0.00451	0.00015	0.00002	0.00450	0.00014	19.53235	4.41954
Fujian	0.00045	0.00006	0.00755	0.00051	0.00005	0.00691	0.00178	23.29297	4.82628
Jiangxi	0.00022	0.00002	0.00388	0.00021	0.00001	0.00355	0.00212	25.25358	5.02529
Shandong	0.00082	0.00073	0.02710	0.00039	0.00022	0.01474	0.00073	357.04299	18.89558
Henan	0.00013	0.00006	0.00777	0.00010	0.00005	0.00676	0.00052	76.89721	8.76911
Hubei	0.00066	0.00009	0.00961	0.00064	0.00007	0.00852	0.00061	40.09426	6.33200
Hunan	0.00127	0.00011	0.01051	0.00139	0.00011	0.01031	0.00172	103.22381	10.15991
Guangdong	0.00045	0.00181	0.04251	0.00045	0.00174	0.04169	0.00053	129.27407	11.36988
Guangxi	0.00040	0.00007	0.00836	0.00025	0.00004	0.00611	−0.00003	17.86174	4.22632
Hainan	0.00000	0.00000	0.00000	0.00016	0.00000	0.00089	0.00005	0.08138	0.28527
Chongqing	0.00007	0.00000	0.00109	0.00001	0.00000	0.00024	0.00059	2.22248	1.49080
Sichuan	0.00035	0.00017	0.01307	0.00036	0.00015	0.01243	0.00056	149.20399	12.21491
Guizhou	0.00186	0.00006	0.00762	0.00031	0.00001	0.00301	0.00084	25.19384	5.01935
Yunnan	0.00007	0.00001	0.00342	0.00010	0.00001	0.00352	−0.00002	42.26552	6.50119
Shaanxi	0.00024	0.00001	0.00372	0.00019	0.00001	0.00333	0.00073	8.27122	2.87597
Gansu	0.00200	0.00007	0.00817	0.00017	0.00000	0.00171	0.00001	0.02165	0.14714
Qinghai	0.00007	0.00000	0.00060	0.00183	0.00000	0.00205	0.01404	5.48537	2.34209
Ningxia	0.00151	0.00001	0.00283	0.00139	0.00001	0.00251	0.00146	2.26042	1.50347
Xinjiang	0.00000	0.00000	0.00000	0.00051	0.00002	0.00492	0.00116	31.90758	5.64868
**Average**	**0.00060**	**0.00015**	**0.00822**	**0.00052**	**0.00012**	**0.00709**	**0.00119**	**46.45210**	**5.45703**

**Table 6:** The Second Part of Forecasting performance of LS-SVM-FM (3).

	Number of Health Examinations (10,000 persons)			Patients Discharged (10,000 persons)		
	MPE	MSE	SDE	MPE	MSE	SDE
China	0.05829	1,574,796.96650	1254.90915	0.00024	29,753.99626	172.49347
Beijing	0.05383	2194.24540	46.84277	0.00205	26.98889	5.19508
Tianjin	0.00539	44.62784	6.68041	0.00024	1.53127	1.23745
Hebei	0.10162	3338.60733	57.78068	0.00107	98.52758	9.92611
Shanxi	0.93100	264,433.55297	514.23103	0.00011	24.02664	4.90170
Inner Mongolia	0.00000	0.00000	0.00016	0.00037	6.94152	2.63468
Liaoning	0.01432	879.38933	29.65450	0.00036	43.02915	6.55966
Jilin	0.08450	532.94037	23.08550	0.00120	15.60832	3.95074
Heilongjiang	0.15653	1442.59133	37.98146	0.00212	77.74058	8.81706
Shanghai	0.35348	4291.54063	65.50985	0.00007	2.18768	1.47908
Jiangsu	0.58290	18,198.78578	134.90288	0.00000	0.00001	0.00307
Zhejiang	0.56100	17,154.77353	130.97623	0.00022	21.99573	4.68996
Anhui	0.10377	2265.71143	47.59949	0.00016	22.42324	4.73532
Fujian	0.09960	1620.21954	40.25195	0.00111	23.02546	4.79849
Jiangxi	0.02990	418.17833	20.44941	0.00379	87.44515	9.35121
Shandong	0.05872	8440.11959	91.87012	0.00079	390.75954	19.76764
Henan	0.09288	5285.62259	72.70229	0.00044	71.24126	8.44045
Hubei	0.02366	925.55176	30.42288	0.00067	47.80071	6.91381
Hunan	0.02203	868.42598	29.46907	0.00159	102.13969	10.10642
Guangdong	0.18586	78,816.83292	280.74336	0.00051	175.82555	13.25992
Guangxi	0.02157	688.26442	26.23479	−0.00005	24.98931	4.99893
Hainan	0.26425	53.91894	7.34295	0.00006	0.07449	0.27293
Chongqing	0.02812	453.15321	21.28740	0.00070	2.61682	1.61766
Sichuan	0.02669	2253.60230	47.47212	0.00064	151.31722	12.30111
Guizhou	0.00000	0.00000	0.00001	0.00078	25.06643	5.00664
Yunnan	0.00858	350.20840	18.71386	0.00005	48.79165	6.98510
Shaanxi	0.13403	1985.82231	44.56257	0.00071	8.36057	2.89147
Gansu	0.01780	182.81707	13.52099	0.00009	0.46562	0.68236
Qinghai	0.08828	25.76667	5.07609	0.00148	0.73092	0.85494
Ningxia	0.01259	13.25353	3.64054	0.00149	2.46583	1.57030
Xinjiang	0.05300	1411.59057	37.57114	0.00126	33.53680	5.79110
**Average**	**0.13720**	**13,952.33714**	**62.88588**	**0.00080**	**51.25512**	**5.65801**

## Data Availability

Not applicable.

## Supplementary Materials

The following supporting information can be downloaded at https://www.mdpi.com/article/10.3390/ijerph20021381/s1. Table S1: main variables of the SBM to calculate the regional eco-efficiency; Figure S1: the number of visits of outpatient emergency departments by sub-department in hospitals in China; and Figure S2: the number of visits of outpatient emergency departments by sub-department in hospitals in China.
