# Supplementary material for: Can Regional Eco-Efficiency Forecast the Changes in Local Public Health: Evidence Based on Statistical Learning in China
Source: Int J Environ Res Public Health. 2023 Jan 12;20(2):1381. doi: 10.3390/ijerph20021381 (PMC9859319; doi:10.3390/ijerph20021381)
Supplement: Supplementary file 1 [file ijerph-20-01381-s001.zip › supplementary material.pdf]

Table S1. Main variables of the SBM to calculate the regional eco-efficiency

**Categories**		**Names**	**Measure**	**Descriptive Statistics**					
				**Max**		**Min**		**Average**	**Standard Deviation**
**Inputs**	**Main resource inputs**	Total Consumption of Energy	Total Consumption of Energy (10,000 tons of Standard Coal Equivalent)	38899.00	Shandong (2012)	407.00	Hainan (1998)	10399.66	7726.82
	**Undesirable inputs**	Pullutant Emission in Waste Gas	Sulphur Dioxide by Region (tons)	1760057.00	Shandong (1998)	9832.01	Beijing (2017)	566453.24	376716.51
		Pullutant Emission in Waste Water	Total Waste Water Discharged by Region (10,000 tons)	305543.36	Jiangsu (2015)	3453.00	Qinghai (2013)	80434.44	68432.76

		Industrial Solid Wastes	Industrial Solid Wastes Produced by Region (10,000 tons)	45625.01	Hebei (2012)	68.94	Hainan (1999)	6734.58	7134.06
**Outputs**	**Main Outputs**	Increased value of industrial development	Industrial added value (100, million yuan)	36477.81	Guangdong (2017)	59.09	Hainan (1998)	5185.23	6182.04

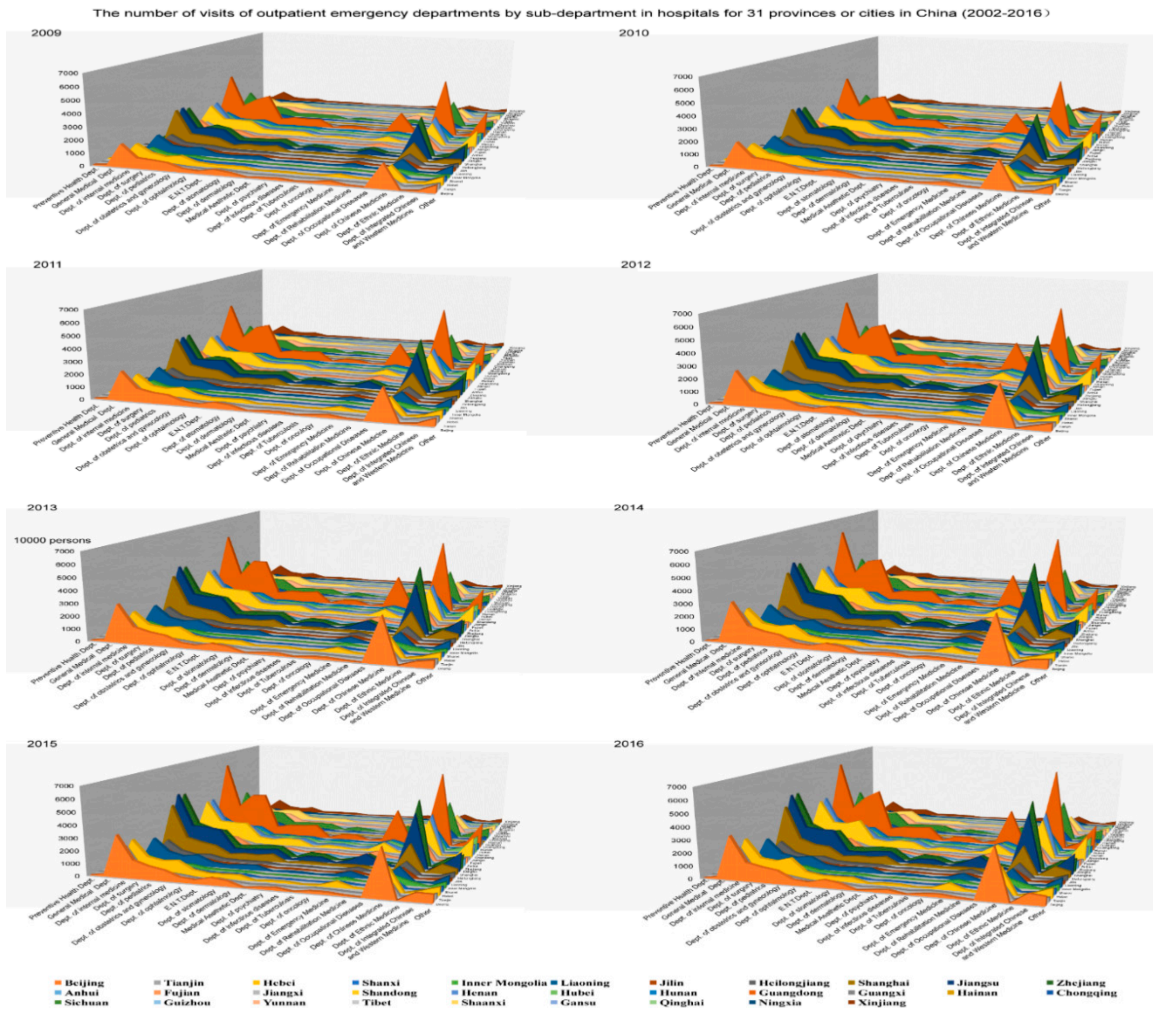

Figure S1. The number of visits of outpatient emergency departments by sub-department in hospitals in China

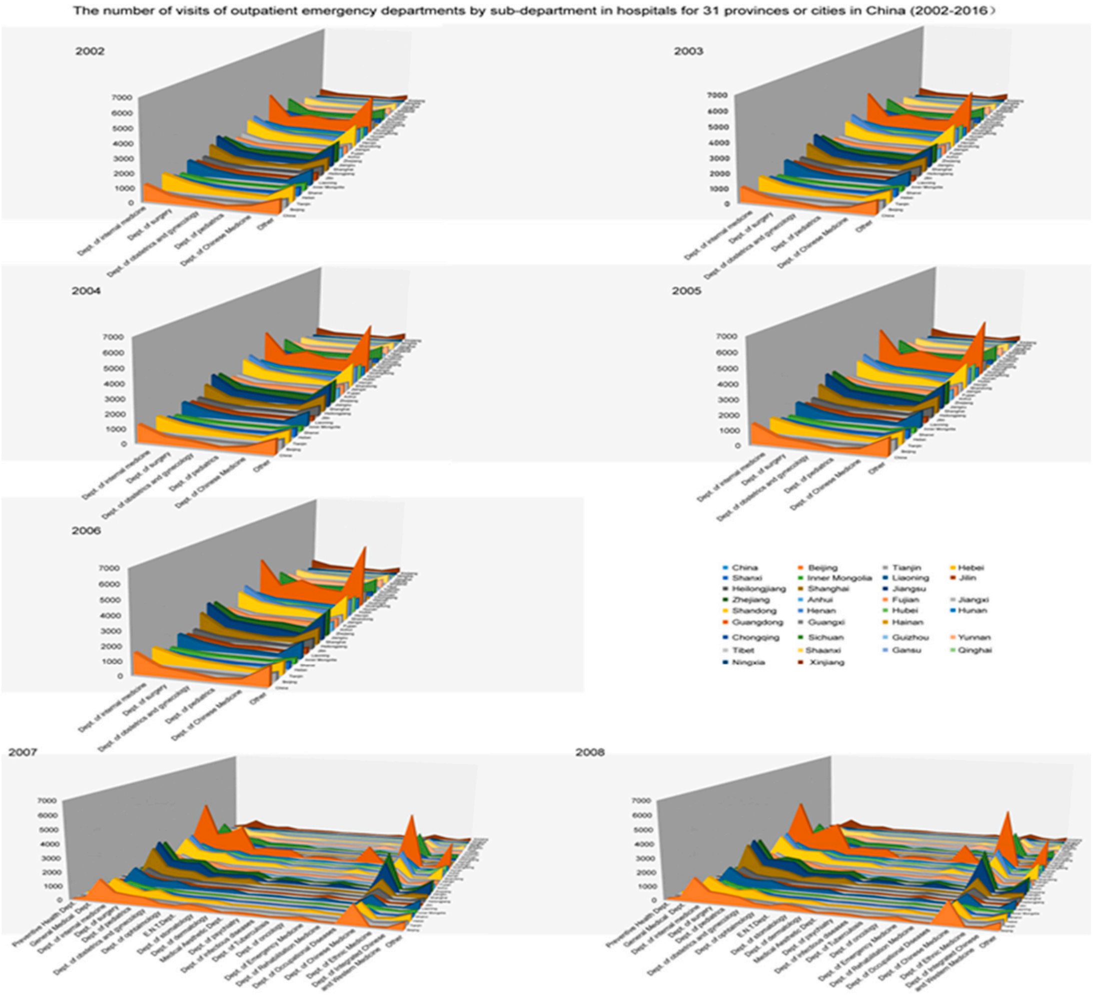

Figure S2. The number of visits of outpatient emergency departments by sub-department in hospitals in China
